# Supplementary material for: Spatial Variation in Stream-Water Bacterial Communities Across Sites Differing in Wild Chinese Giant Salamander Detection Frequency
Source: Biology (Basel). 2026 Sep 18;15(18):1647. doi: 10.3390/biology15181647 (PMC13604818; doi:10.3390/biology15181647)
Supplement: Supplementary file 1 [file biology-15-01647-s001.zip › Data Related to “Spatial Variation in Stream-Water Bacterial Communities across Sites Differing in Wild Chinese Giant Salamander Detection Frequency”/Microbial Data/result/03.TaxonVisual/heatmap/sample/heatmap.sample.g.pdf]

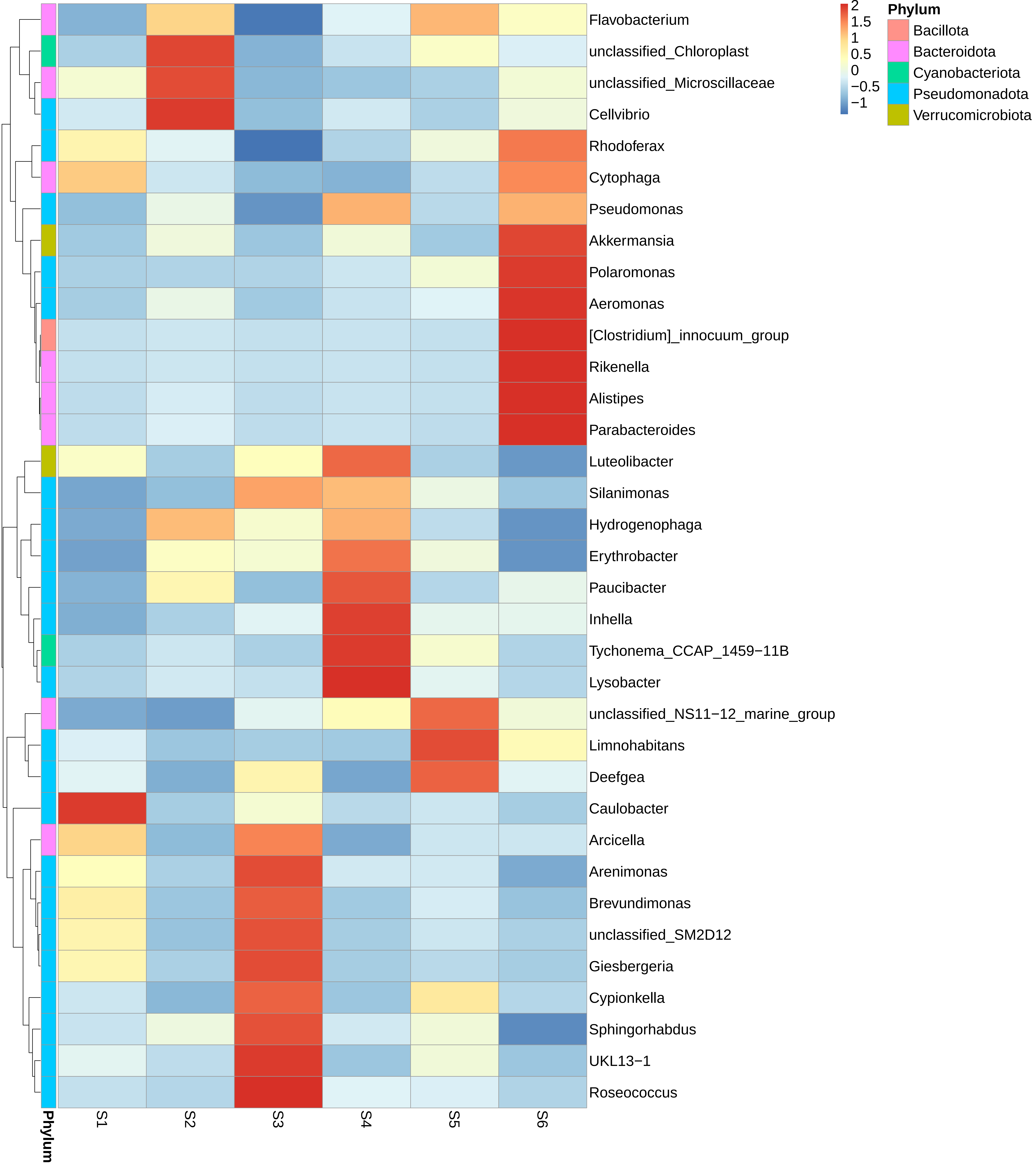

Flavobacterium
unclassified_Chloroplast
unclassified_Microscillaceae
Cellvibrio
Rhodoferax
Cytophaga
Pseudomonas
Akkermansia
Polaromonas
Aeromonas
[Clostridium]_innocuum_group
Rikenella
Alistipes
Parabacteroides
Luteolibacter
Silanimonas
Hydrogenophaga
Erythrobacter
Paucibacter
Inhella
Tychonema_CCAP_1459−11B
Lysobacter
unclassified_NS11−12_marine_group
Limnohabitans
Deefgea
Caulobacter
Arcicella
Arenimonas
Brevundimonas
unclassified_SM2D12
Giesbergeria
Cypionkella
Sphingorhabdus
UKL13−1
Roseococcus
S1
S2
S3
S4
S5
S6
Phylum
2
1.5
1
0.5
0
−0.5
−1
Phylum
Bacillota
Bacteroidota
Cyanobacteriota
Pseudomonadota
Verrucomicrobiota
